# Long-Term Effects of Attentional Performance on Functional Brain Network Topology

**DOI:** 10.1371/journal.pone.0074125

**Published:** 2013-09-09

**Authors:** Thomas P. K. Breckel, Christiane M. Thiel, Edward T. Bullmore, Andrew Zalesky, Ameera X. Patel, Carsten Giessing

**Affiliations:** 1 Biological Psychology Lab, Department of Psychology, University of Oldenburg, Oldenburg, Germany; 2 Brain Mapping Unit, Behavioural and Clinical Neuroscience Institute, University of Cambridge, Cambridge, United Kingdom; 3 Clinical Unit Cambridge, GlaxoSmithKline, Addenbrooke’s Centre for Clinical Investigations, Cambridge, United Kingdom; 4 Cambridgeshire & Peterborough NHS Foundation Trust, Cambridge, United Kingdom; 5 National Neuroscience Facility, University of Melbourne, Melbourne, VIC, Australia; Beijing Normal University, Beijing, China

## Abstract

Individuals differ in their cognitive resilience. Less resilient people demonstrate a greater tendency to vigilance decrements within sustained attention tasks. We hypothesized that a period of sustained attention is followed by prolonged changes in the organization of “resting state” brain networks and that individual differences in cognitive resilience are related to differences in post-task network reorganization. We compared the topological and spatial properties of brain networks as derived from functional MRI data (N = 20) recorded for 6 mins before and 12 mins after the performance of an attentional task. Furthermore we analysed changes in brain topology during task performance and during the switches between rest and task conditions. The cognitive resilience of each individual was quantified as the rate of increase in response latencies over the 32-minute time course of the attentional paradigm. On average, functional networks measured immediately post-task demonstrated significant and prolonged changes in network organization compared to pre-task networks with higher connectivity strength, more clustering, less efficiency, and shorter distance connections. Individual differences in cognitive resilience were significantly correlated with differences in the degree of recovery of some network parameters. Changes in network measures were still present in less resilient individuals in the second half of the post-task period (i.e. 6–12 mins after task completion), while resilient individuals already demonstrated significant reductions of functional connectivity and clustering towards pre-task levels. During task performance brain topology became more integrated with less clustering and higher global efficiency, but linearly decreased with ongoing time-on-task. We conclude that sustained attentional task performance has prolonged, “hang-over” effects on the organization of post-task resting-state brain networks; and that more cognitively resilient individuals demonstrate faster rates of network recovery following a period of attentional effort.

## Introduction

Many daily activities require the maintenance of attention over long periods of time which causes behavioural performance to deteriorate [1]–[3]. Performance decline comprises slowing of signal detection and information processing, behavioural changes often described as vigilance decrements [2]. In contrast, individuals whose performance declines less rapidly as a function of time on task can be described as cognitively resilient. In real life, humans recover from vigilance decrements during rest periods (e.g. between working shifts) with low cognitive demands. However, it remains an open question whether long-lasting effects of sustained attention following task performance reflect on-going changes in the functional organization of resting state networks. It is also not known if individual differences in cognitive resilience are related to differences in post-task network reorganization.

There has been recent progress to describe the human brain as a complex network of interconnected processing nodes [4], [5]. Based on graph analytical approaches, previous studies documented that variations of functional network integration and the topological efficiency of information transfer are correlated with behavioural performance [6], [7]. Studies investigating effects of ageing documented that older people show poorer attentional performance [8], [9], and less integrated brain networks with higher clustering and network cliquishness [10], [11]. Similar results were shown for ADHD children [12] and patients with Alzheimer’s disease [13].

Previous studies documented the flexibility of the brain network topology [14]. It was shown that the integration of brain networks dynamically varied with different levels of task difficulty. However, there is also support for the principle that the flexibility of the brain is limited and that the processing of demanding tasks has long-lasting impact on neural activations and brain networks, possibly reducing the brain’s capacity to adapt to new task demands. Duff et al. [15] and Waites et al. [16] compared resting state (RS) fMRI data before and after task performance and reported changes in functional connectivity directly following task performance. Further studies have shown that task induced learning can modulate subsequent RS activity in specific task relevant networks [17]–[19]. Results of Barnes et al. [20] revealed that endogenous neural oscillations in local brain regions change after a demanding task and need more than 400 sec to begin to recover. Thus, there are hints that task performance can modulate “resting state” dynamics and networks in post-task fMRI data.

We acquired fMRI data during RS periods before and after a vigilance task and used a graph analytical approach to investigate long-lasting changes in functional brain topology induced by sustained attention. We assumed that (i) prolonged attentional performance leads to less integrated networks with more clustered brain regions and a change from long-distance to short-distance connections. Furthermore, we expected (ii) that altered network attributes recover slowly after task processing and that (iii) network recovery and individual task performance are correlated.

To further investigate possible mechanisms underlying these long-lasting changes in network topology we also analyzed changes in network topology during task performance. Our own work suggests that cognitive fatigue and prolonged attentional performance affect endogenous neural resting state activations [21]. Other studies revealed that endogenous neural activations persist during task performance and contribute to the prediction of behavioural performance [22]. Thus, we hypothesized that the disintegration of endogenous brain networks already started during task processing and that this process continued during following resting state periods (especially in subjects with low cognitive resilience and lower task performance). To investigate endogenous changes in brain network topology which were not directly correlated with changes in external stimulation we compared different time periods of task processing in which the participants performed the same task and in which their brain system was externally pertubated with identical external stimulations.

## Materials and Methods

### Subjects

Twenty healthy, right-handed subjects (11 female, 9 male; mean age = 27.0 years, range 24 to 39 years) participated in the experiment.

The study was approved by the ethics committee of the German Psychological Association (http://www.dgps.de/, PI: Christiane Thiel, registration number: CT05022008DGPS) and subjects signed written informed consent.

### Experimental Design

**Figure 1:**
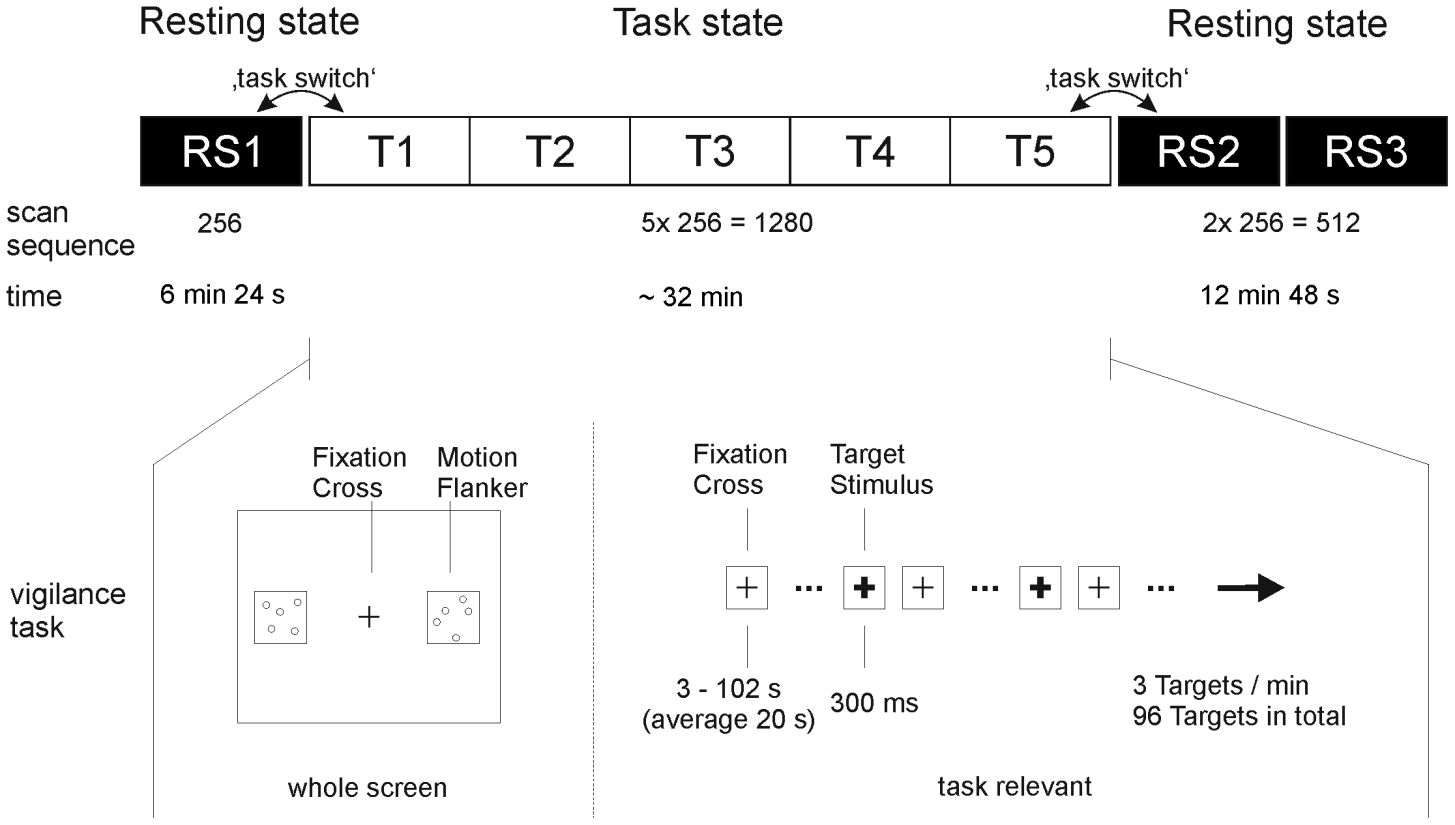
Experiment design. **Top:** Order of the resting state (RS) and task periods (T) with the number of acquired whole brain scans. The task was separated from the resting state periods by brief instructions. Task periods (T1 to T5) were continuously measured and later split up into five scan periods with the same length. The post-task resting state period (RS2 and RS3) was also measured continuously and split up later for the analyses. The changes from the first resting state period (RS1) to the first task period (T1) as well as from the last task period (T5) to the first post-task resting state period (RS2) are referred to as ‘task switch’. **Bottom:** Scheme of the vigilance task during the task period. Participants were instructed to detect a red fixation cross (here depicted in bold).

Each scanning session was divided into different time periods in which either resting state (RS) or BOLD activations during task processing were measured (see Figure 1). There were one RS block before the task with 256 scans and one RS block following the task with 512 scans. The second RS block was divided into two halves so that in total three RS periods were analysed with 256 scans each (equivalent to 6 min 24 s). During RS data acquisition subjects were instructed to fixate a black fixation cross that was presented in the centre of a light grey background. Studies which compared RS periods with eyes open and eyes closed found differences in low frequency BOLD fluctuations and connectivity measures within visual brain areas [23], [24]. In the current study we instructed subjects to keep their eyes open in order to have similar visual stimulation during the task and resting state conditions (see also [23], p. 680).

### Vigilance Task

During the task period, subjects performed a visual vigilance task in which a display with a central black fixation cross (the same as used during the RS condition) and two additional flanker windows were presented (Figure 1). Subjects were instructed to detect a colour change of the central fixation cross from black to red for 300 ms by pressing a response button as fast as possible. In total 96 targets were presented in 32 min. The targets were equally distributed over the experiment by presenting three target stimuli per minute (with random interstimulus intervals within each minute). In the flanker windows, five dots randomly moved with low speed or were kept still. Both phases alternated every 24 s. Subjects were instructed to ignore the flanker windows.

To investigate changes in network topology during task processing fMRI scans acquired during task performance were divided into blocks of 256 scans each, building five different task blocks (T1 to T5). Within these task blocks subjects processed the same trials with the same number of targets and the same number of alternating “motion on” and “motion off” phases in the flanker windows. The analysis of local task activations related to specific task conditions were published previously [25].

### Analysis of the Behavioural Data

The individual vigilance decrement of each subject was estimated by the slope of a robust linear regression analysis with the individual response latencies or reaction times (RTs) as dependent variable and the series of attentional trial numbers as independent variable. In contrast to standard linear regression analysis in which each data point has equal influence on the least square curve fit, robust regression analysis uses a bisquare weighting function so that data points further apart from the expected line get reduced weight. Robust fits are less affected by outliers [26]. To take the between subject variance into account, the twenty estimated slopes (one per subject) were further investigated within a second-level one-sample t-test to show that the mean slope averaged over subjects was different from zero [27].

The Stanford Sleepiness Scale (SSS) [28] was administered before and after the fMRI experiment (outside the scanner) to measure subjective feelings of fatigue on a 10-point scale. Individual SSS scores were compared (before vs. after the fMRI experiment) using two-tailed paired t-tests.

### FMRI Data Acquisition

Functional and structural images were acquired on a 1.5 Tesla MRI scanner (Siemens MAGNETOM Sonata, Siemens AG, Erlangen, Germany). Functional images were obtained using multislice T2*-weighted gradient echo planar imaging (EPI). Each volume consisted of 17 axial slices (voxel size of 3×3 mm, 4 mm in slice thickness, slice gap of 1.6 mm, field of view (FoV) = 200×200 mm^2^, relaxation time (TR) = 1500 ms, echo time (TE) = 50 ms and 90° flip angle). EPI data were continuously measured with the same sequence in all RS and task periods. Structural T1-weighted images were obtained after the fMRI experiment, using magnetization frequency pulse and rapid gradient-echo (MP RAGE) sampling: 1 mm isotropic voxels, 176 slices, FoV = 256×256 mm^2^, TR = 2130 ms, TE = 3.93 ms and 15° flip angle.

### FMRI Data Processing and Time-series Analysis

**Figure 2:**
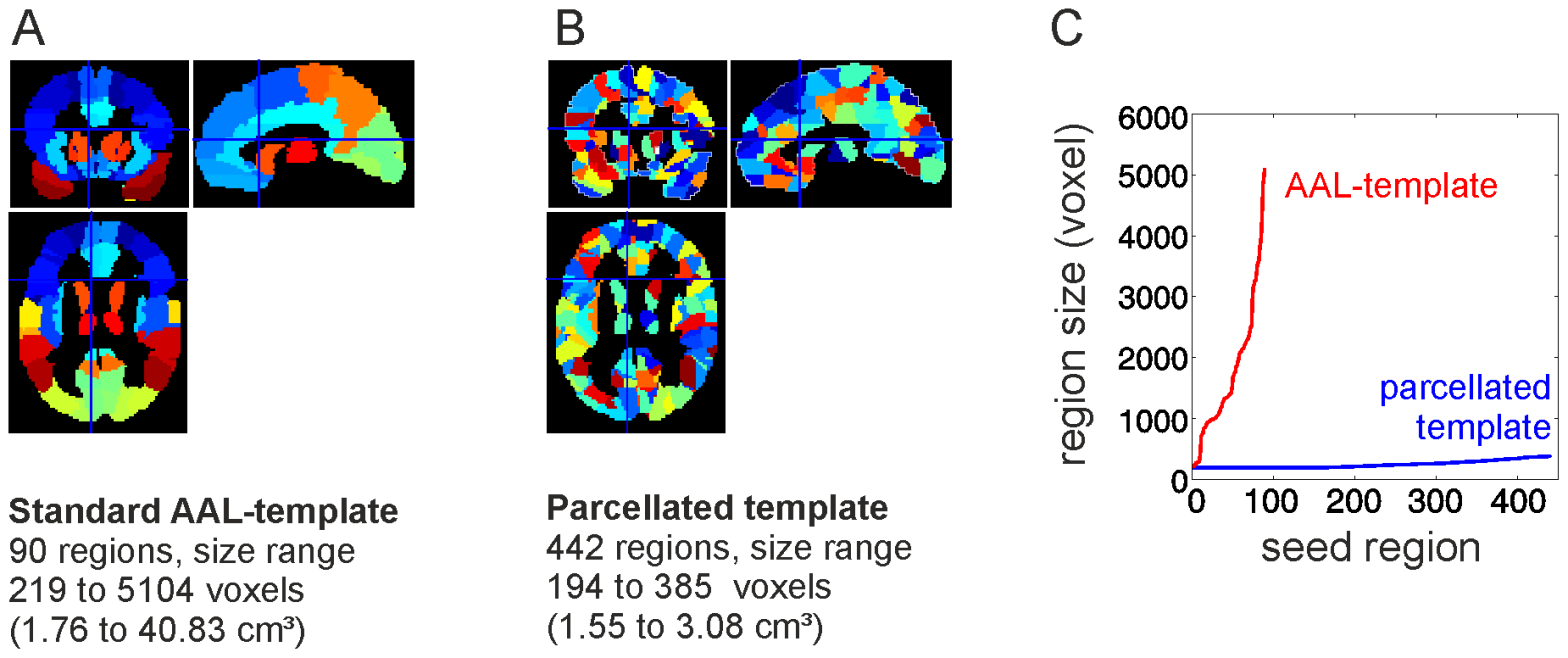
Illustration of parcellation routine for seed region/node definition. Cortical and subcortical brain regions were parcellated into 442 brain nodes. (**A**) The parcellation routine was based on an anatomically parcellated and labeled T1 volume provided by the AAL toolbox (Tzourio-Mazoyer et al., 2002). (**B**) The parcellation routine randomly and homogenously split the larger AAL regions into 442 smaller subparcels while preserving the macro-anatomical borders as defined in the AAL-template. (**C**) This parcellation approach creates more homogenous seed region sizes as compared to the original AAL-template so that possible biasing effects of parcel sizes on functional connectivity (Salvador et al., 2008; Fornito et al., 2010) were prevented. The main statistical analyses were repeated with alternative templates in order to assure that the reported effects of the RS data analysis were not driven by a singular parcellation template (see File S1).

The time series of each voxel was corrected for head motion and slice timing offsets using SPM8 (http://www.fil.ion.ucl.ac.uk/spm/). The functional images were spatially normalized to standard stereotaxic MNI space (Montreal Neurological Institute; http://www.mni.mcgill.ca/) and regionally parcellated using a template image comprising 442 cortical and subcortical brain regions to estimate the mean fMRI time series for each region for each participant during each RS and task period [29], [30]. In order to avoid possible biasing effects of parcel sizes on functional connectivity [31], [32], we ensured that the range in size of the 442 nodes generated by sub-parcellating the AAL template was substantially smaller (1.55–3.08 cm^3^;) than the range in size of the 90 nodes of the template (1.76–40.83 cm^3^, regions of cerebellum were excluded; see Figure 2). Note that we performed the main statistical analyses of the resting state periods also with alternative templates in order to assure that our main findings were not driven by the choice of a single parcellation template (see File S1).

To reduce motion effects motion parameters of the spatial realignment were regressed out from the mean time series. Previous studies documented the behavioural relevance of graph metrics based on fMRI signal oscillations in the frequency range just below 0.1 Hz [33]–[35]. Therefore, each regional time series was band-pass filtered (wavelet scale 3: 0.042–0.083 Hz) using the maximum overlap discrete wavelet transform (MODWT) [36]. For each pair of nodes the wavelet correlation was estimated and the absolute correlation values resulted in a 442 by 442 association matrix for each subject, RS and task period. The global strength of connectivity was computed as the mean of each association matrix. To compute the nodal connectivity strength of a particular node we averaged the functional connectivity between this node and all other nodes within the network.

### Topological Metrics of Brain Graphs

Binary graphs of different connection densities were constructed based on the association matrix. A thresholding algorithm starting with the minimum spanning tree (MST) was used to ensure that each node was connected with at least one other node even at the sparsest connection density. Following the MST the algorithm adds the highest correlations of each node in an iterative way (see [37] for further information). Graphs at 15 different costs levels were constructed for each subject and RS period in the range of 2.5%–50%, with smaller sampling intervals in the lower cost range. The connection density defines the number of edges in a graph expressed as a ratio to the maximum possible number of edges (N*(N-1)/2 = 97,461). In agreement with previous studies on functional brain topology (e.g. [38], [39]) we stopped at 50% connection density since connections at higher costs are likely to be non-biological and influenced by noise. For the statistical analyses topological measures were calculated for each cost level separately and averaged over the entire cost range (see below). We averaged graph metrics across the entire cost range to avoid multiple comparisons at individual sampling points and to reduce the dependency of any significant differences in network topology on the arbitrary choice of a single connection density. This approach has been used in previous graph analyses [38], [39] and its theoretical background was discussed in detail by Ginestet et al. [40].

Based on these adjacency matrices we calculated the central graph theoretical measures of global, nodal and local efficiency following the formulas of Latora and Marchiori [41], [42]. These metrics base on the minimum path lengths between connected nodes. The nodal efficiency of a particular node is inversely related to the mean minimum path length between this node and the rest of the network. In contrast, global efficiency is an estimate for the efficiency of an entire network, and is the mean over the nodal efficiencies. Thus networks with high global efficiency have highly integrated organization, characterized by short minimum path length between any pair of regional nodes.

Local efficiency is closely related to the clustering coefficient [43], and reflects the network’s capacity for information transfer between the nearest neighbours of a particular node. This can be averaged to get an estimate of local efficiency for the graph as a whole. Thus networks that have a cliquish organization, characterized by many connections between the nearest neighbours of any given node, will have high local efficiency or clustering. To avoid terminological confusion with global and nodal efficiency, we will refer to this metric here as a measure of clustering.

### Physical Distances

The physical distances were based on the Euclidean distances between the centres of coordinates (MNI-space) of all functionally connected brain seed regions. The averaged physical distances of all nodes built the measure of mean distances. Further, the number of connections/edges in each of several distance bins in the histogram of connection distances was counted (in bin intervals of 5 mm steps, starting with the minimal distance of 3 mm) at the cost level of 50%. Differences between the numbers of edges within each bin at different RS periods were tested with two-sided paired t-tests (Bonferroni corrected).

### Statistical Analysis of Network Metrics

For each network metric we used three different linear mixed-effects models to analyse the data. All models included an intercept term for each subject to model a random offset [44]. Within the *first* mixed-effects model we compared the resting state data before and following task processing and modelled the data with the factor RS period (three levels, RS1, RS2, and RS3), the behavioural performance as continuous factor (the individual vigilance decrements), and the interaction between both factors. In the *second* analysis we compared the change in topology during task processing. Therefore we modelled the network metrics as linear function of time (factor ‘task period’). In a *third* analysis we analyzed the change in network topology from rest to task performance in the beginning of the task period and from task to rest following the task period. The mixed-effects models included the factor ‘task switch’ (the change from RS to task period), the factor ‘time point’ (task switch at the beginning or following the task period), and their interactions.

The network metrics global efficiency, clustering, and mean distances were averaged over the a-priori-defined cost range for both models in order to generalize the results across cost level (see above). In contrast to the normal analyses of variance, linear mixed-effects models can handle data with unequal variances. Thus, before significance testing, variances of data between RS periods were compared and significant in-homogeneities of variances were explicitly modelled within the data analysis.

Pair-wise differences between RS periods were tested post-hoc by two-sided one-sample t-tests. Pearson’s correlations were used to test whether participants with higher or lower vigilance decrements show different changes in network metrics. To simplify this analysis, we also divided the participants into the two subgroups of “attentionally impaired” (subjects with a significant vigilance decrement, n = 10) and “attentionally resilient” subjects (subjects with no significant vigilance decrement, n = 10, see Results: behavioural data) and compared both groups with two-sample t-tests.

To control type 1 error in the context of the multiple comparisons entailed by analysis of nodal efficiency and clustering, we used p≤0.0025 (1/N) as the threshold for significance, so that less than one false positive test is expected for each whole brain analysis of nodal network properties.

## Results

### 1. Behavioural Data

**Figure 3:**
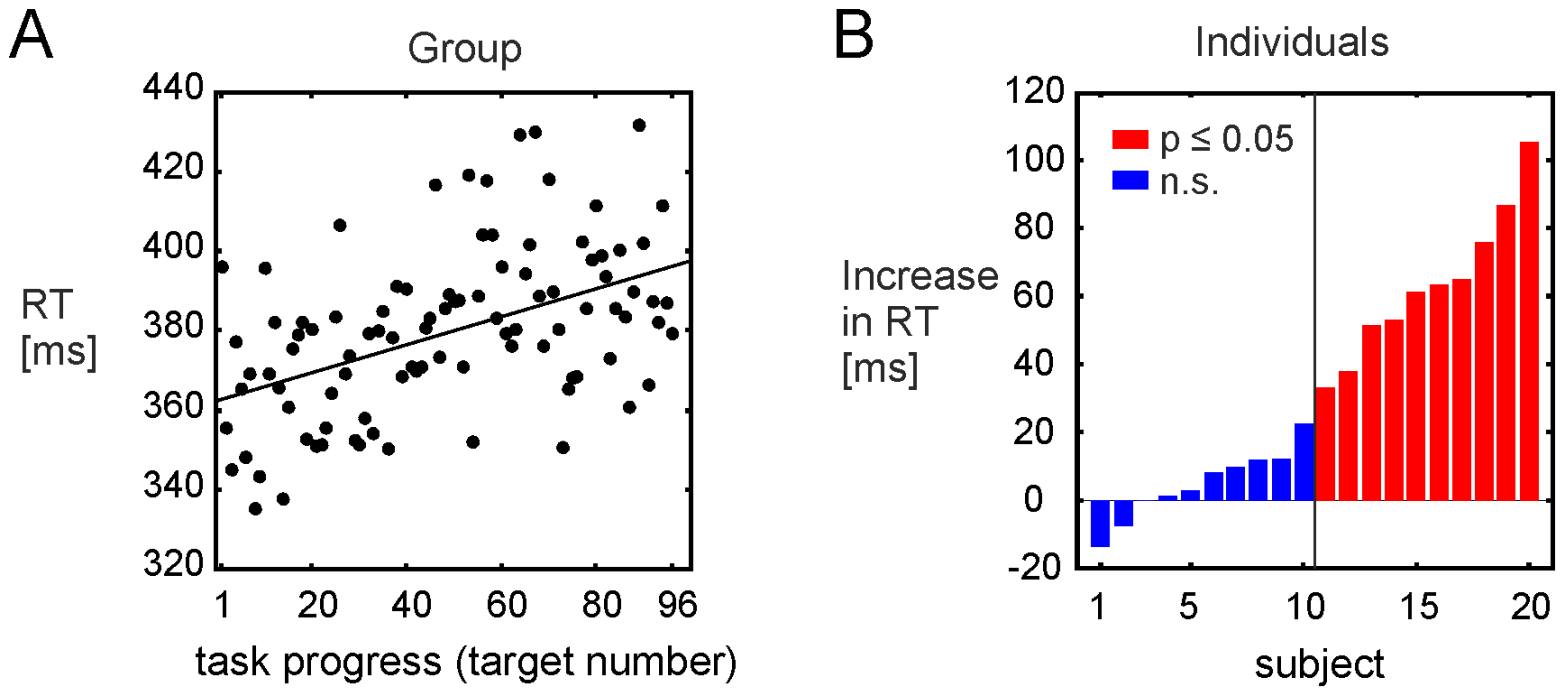
Vigilance decrements in the attentional task. (**A**) Median reaction times averaged across subjects for each of the 96 target presentations during the task are shown. Solid line: The robust fit regression showed a significant RT increase/vigilance decrement. (**B**) On the individual level, participants showed a large variability in their change in RTs during task performance. For each subject, the individual robust fits for the RTs were used to estimate the RT change at the end of the task. For illustration we divided the participants into a group with significant RT increase (red, referred to as ‘attentional impaired’ subjects) and without a significant RT increase (blue, referred to as ‘attentional resilient’ subjects). However, to test the correlation between network parameters and behavioural performance without loss of information, the individual RTs increases were used and tested for a linear relationship.

Subjects generally performed the task well and showed low rates of misses (mean = 1.95, STD = ±0.94). Therefore the analysis of the behavioural data focused on reaction times (RT) only. Reaction times showed a significant vigilance decrement over all subjects (regression on median RTs averaged across subjects; r = 0.48, p≤0.001, Figure 3 A); median RT increased by 33.98 ms at the end of the experiment. A two tailed t-test over the individual regression slopes (robust fit prediction) as a measure of cognitive resilience of each subject confirmed this result (t(19) = 4.41, p<0.001).

However, response latency data also showed high between-subject variability. Ten participants demonstrated a significant vigilance decrement (prolongation of response time for target stimuli presented later in the series); whereas the other ten participants had no significant change in response time to targets over the course of the task (Figure 3 B). We refer to these sub-groups as the ‘attentionally impaired’ and ‘attentionally resilient’ groups, respectively.

The ratings in the Stanford Sleepiness Scale before and after the experiment showed that after task performance participants felt more fatigued (t(19) = 7.89, p≤0.001, mean increase = 2.23, STD = ±1.25). There was no correlation between individual RT decrements and sleepiness ratings.

### 2. Network Metrics

#### 2.1. Pre-test: Variance homogeneity

Before the connectivity strength and network metrics were compared in different experimental conditions it was tested whether their variances were significantly different (using a likelihood ratio tests comparing models with fixed vs. variable variances; see [44]). For those comparisons with significant differences in variance we explicitly modelled unequal variances in the data analysis. Analyses with significant different variances (p<0.05) are marked in the according tables.

#### 2.2. Changes during RS periods (RS1, RS2 and RS3)

**Figure 4:**
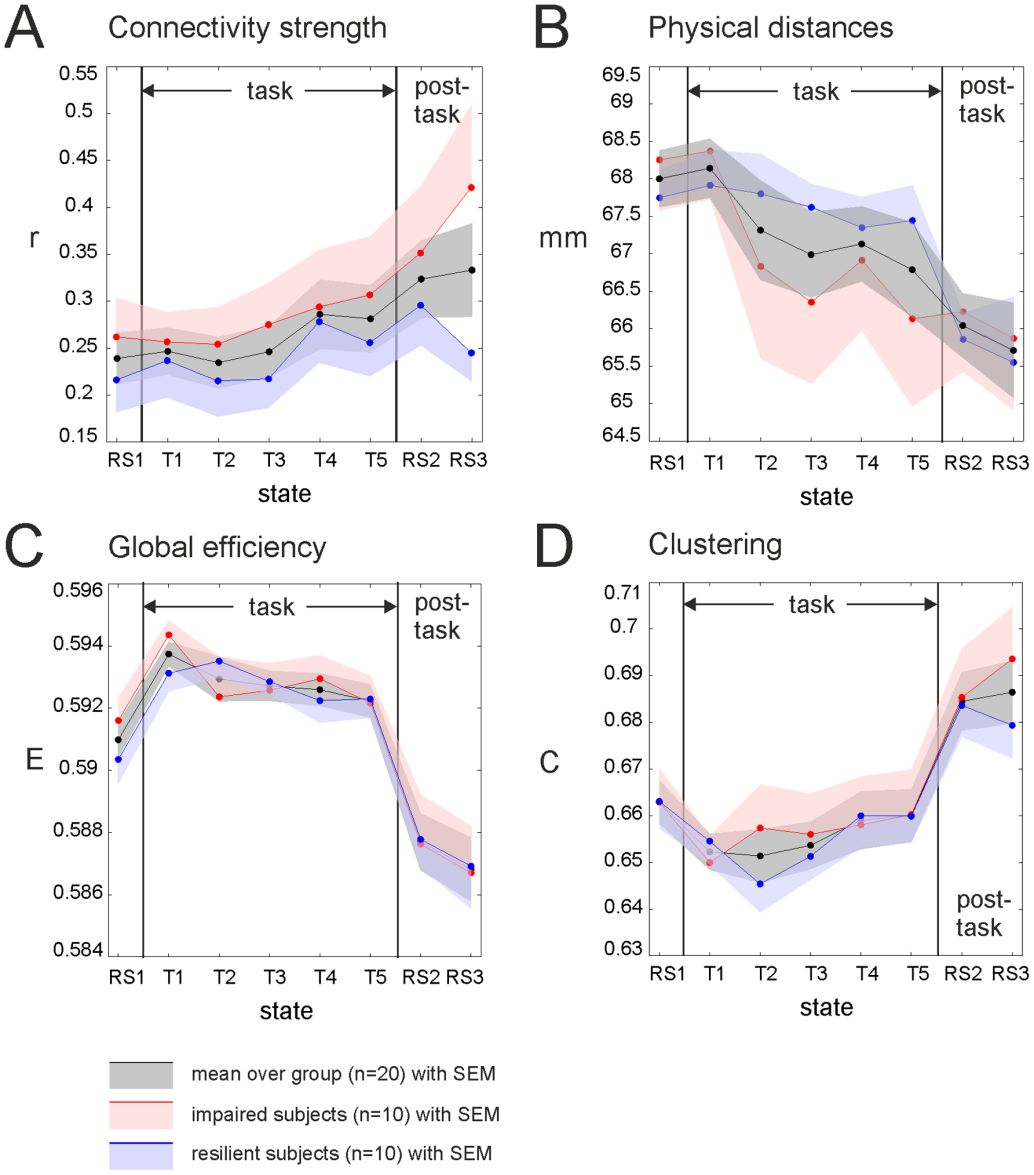
Changes in connectivity strength and network metrics during resting state and task periods. (**A–D**) For better readability the standard error of the mean (SEM) of the two subgroups (impaired vs. resilient subjects) is only depicted one sided. (**A**) Global connectivity strength averaged over all nodes. *Post-task phase*: Across all subjects (black) connectivity strength was significantly increased following the task (RS1 to RS2) and stayed increased during the post-task phase (RS2 to RS3). Attentionally resilient subjects (blue) began to recover during the post-task phase and showed a decrease in functional connectivity in the direction of the pre-task values, whereas attentionally impaired subjects (red) showed further increases in global connectivity strength until the end of the measurement (see also Figure 5 A). *During task*: Connectivity continuously increased during the task. Further, the overall level of connectivity strength during the task (as averaged over all task periods) was significantly correlated with individual performance. Subjects with higher vigilance decrements (higher latencies in reaction times towards the end of the task) showed higher global connectivity strengths. (**B–D**) Graph metrics were averaged over the entire investigated cost range (see File S2 for results at single selected cost levels) (**B**) Mean physical connection distances in mm. *Post-task phase*: Physical distances were significantly reduced following task performance and stayed low in the second post-task phase (RS3) as compared to the first resting state period (RS1) (see also Figure 5 D for changes in connection lengths distribution). *During task*: Physical distances continuously decreased during the task over all subjects. (**C**) Global efficiency. *Post-task phase*: Global efficiency was significantly reduced following task performance as compared to the first RS period. Global efficiency did not change within the post-task phase (RS2 to RS3). *During task*: Global efficiency significantly increased at the beginning of the task (RS1 to T1) and decreased at the end of the task (T5 to RS2). Over all task periods (T1 to T5) global efficiency continuously decreased in all subjects. (**D**) Clustering. *Post-task phase*: Clustering was significantly higher following the task as compared to the first RS period. The group average (black) stayed increased in the second post-task period (RS3). Attentionally impaired subjects (red) showed a further increase in clustering in the last RS period, whereas more resilient subjects showed a first recovery or change of clustering in the direction of the pre-task values (see also Figure 5 C). *During task*: Clustering significantly decreased with the start of the task (RS1 to T1) and significantly increased again when the task ended (T5 to RS2). Over the task periods (T1 to T5) clustering continuously increased in all subjects.

*Overall changes following task performance and in the post-task phase.* For all network metrics (connectivity strength, global efficiency, clustering, and physical distances) the data analysis revealed a significant main effect for the factor RS period (Table 1). Immediately following task performance, all network metrics were altered (see post-hoc Table 2): Connectivity strength and clustering were significantly increased, global efficiency and physical distances were significantly reduced following task performance (from RS1 to RS2) (Figure 4). For the graph metrics, the changes following the task were more pronounced in the lower cost range (see Figure S2.1 in File S2) in which connections based on higher correlation values that were less affected by noise.

**Table 1:** Mixed-effects analysis of resting state data: Effects of resting state condition, performance and resting condition-by-performance interaction on (i) mean connectivity strength, (ii) functional network and (iii) physical distance metrics.

Response	Factor	Statistics
Connectivity Strength	**Resting State (RS)**	**F(2,36) = 5.80, p<0.01**
	**Performance**	**F(1,18) = 8.46, p<0.01**
	Interaction RS*Performance	**F(2,36) = 4.32, p<0.05**
Global Efficiency	**RS**	**F(2,36) = 15.8, p<.0001**
	Performance	F(1,18) = 0.1, p = 0.74
	Interaction RS*Perf.	F(2,36) = 1.6, p = 0.22
Clustering(a)	**RS**	**F(2,36) = 8.35, p<0.01**
	Performance	F(1,18) = 2.59, p = 0.13
	Interaction RS*Perf.	**F(2,36) = 6.18, p<0.01**
Physical Distances(a)	**RS**	**F(2,36) = 22.42, p<0.001**
	Performance	F(1,18) = 0.24, p = 0.63
	Interaction RS*Perf.	F(2,36) = 1.82, p = 0.17

(a) corrected for unequal variances.

**Table 2:** Pairwise comparisons of resting state data: Effects on (i) mean connectivity strength, (ii) network topology, (iii) physical distances and (iv) its correlations with performance.

Measure		Task (RS1–RS2)	Post-task (RS2–RS3)	RS1–RS3
Connectivity Strength	Change(a)	**T = −2.69, p<0.02**	T = −0.41, p = 0.69	**T = −2.27, p<0.04**
	Correlation with performance(b)	R = −0.16, p = 0.50	**R = −0.62, p<0.01**	**R = −0.47, p<0.05**
	(and group difference(c))	(T = −0.16, p = 0.87)	**(T = −3.12, p<0.01)**	(T = −1.66, p = 0.12)
Global Efficiency	Change	**T = 3.85, p<0.01**	T = 1.28,p = 0.22	**T = −5.09, p<0.01**
	Correlation with performance	R = 0.18, p = 0.44	R = 0.24, p = 0.32	R = 0.39, p = 0.09
	(and group difference)	(T = 0.82, p = 0.43)	(T = 0.05, p = 0.96)	(T = 0.89, p = 0.38)
Clustering	Change	**T = −3.66, p<0.01**	T = −0.64, p = 0.53	**T = −3.79, p<0.01**
	Correlation with performance	R = −0.08, p = 0.75	**R = −0.63, p<0.01**	R = −0.38, p = 0.10
	(and group difference)	(T = −0.16, p = 0.88)	**(T = −2.23, p<0.05)**	(T = −1.17, p = 0.26)
Physical Distances	Change	**T = 5.41, p<0.001**	T = 0.74, p = 0.46	**T = 4.49, p<0.001**
	Correlation with performance	R = 0.30, p = 0.20	R = 0.16, p = 0. 49	R = 0.36, p = 0.12
	(and group difference)	(T = 0.18, p = 0.86)	(T = 0.05 p = 0.96)	(T = 0.18, p = 0.86)

(a) Change between resting states, paired t-tests, df = 19.

(b) Differences between resting states correlated with individual vigilance decrements.

(c) Group comparison between attentionally impaired and resilient subjects (see text), two-sample t-tests, df = 18.

These changes immediately following the task persisted in the post-task phase (RS2 to RS3) when averaged over the whole group (see relation to performance below) and were still significantly different when compared to the RS period previous to the task (RS1 to RS3) (Table 2, Figure 4).

**Figure 5:**
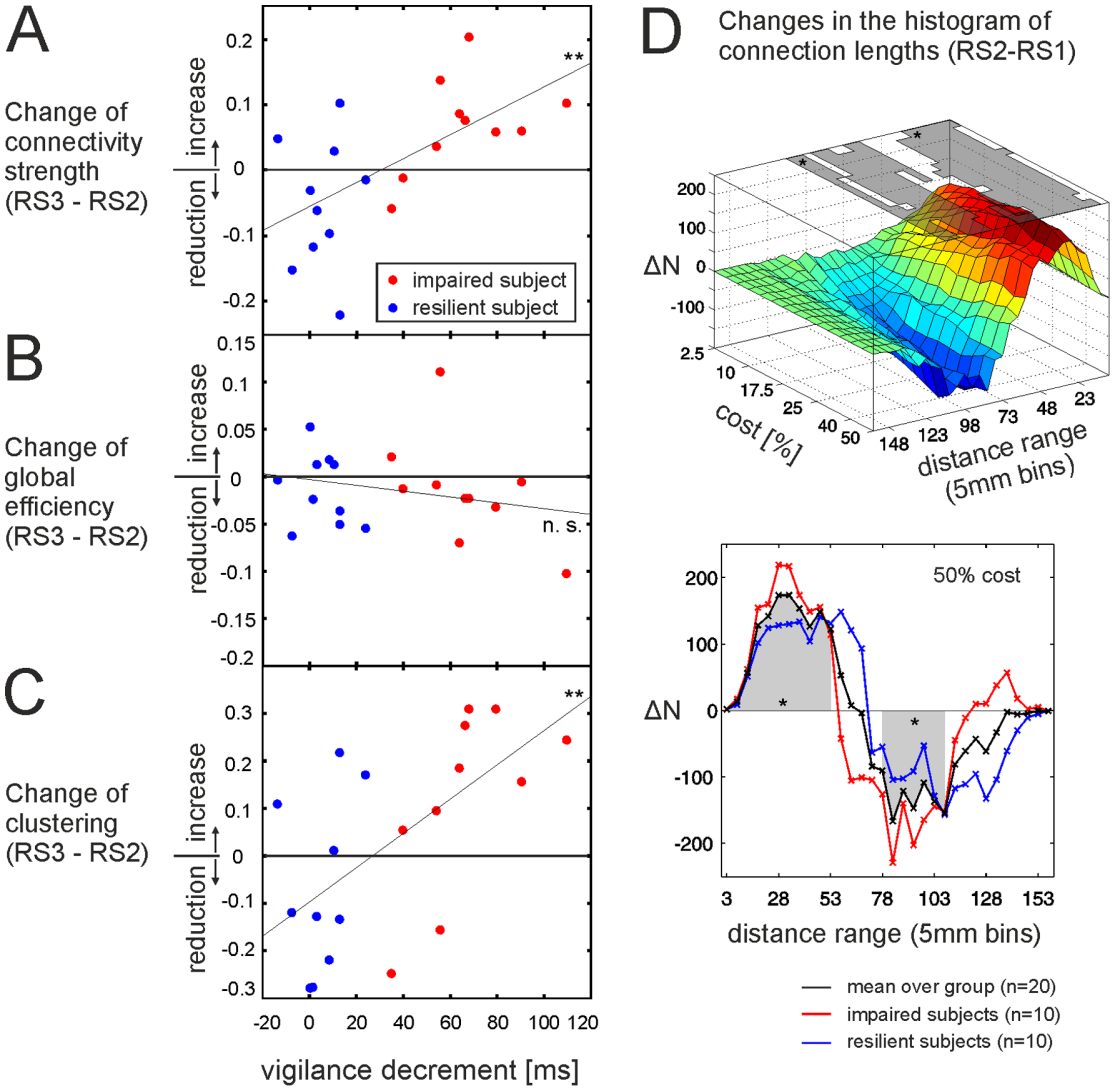
Changes in connectivity strength and network topology during the post-task resting state period (RS2 vs. RS3) and relation with behavioural performance; Changes in the histogram of physical distances. (**A–C**) Correlation between post-task changes (RS3-RS2) and individual performance. (**A**) Connectivity strength: More attentional resilient subjects (blue, subjects with low vigilance decrements) show a reduction in connectivity strength from RS2 to RS3, whereas impaired subjects (red) show a further increase. (**B**) Changes in global efficiency from RS2 to RS3 did not correlate with performance. (**C**) **Changes in** clustering during the post-task phase significantly correlated with individual performance. Resilient subjects (blue) show decreases in clustering from RS2 to RS3, impaired subjects (red) further increase in clustering. (**D**) Change in histogram of mean distances between nodes following task performance (RS1 vs. RS2): The number of connections were counted in 32 distance bins of 5 mm (ΔN: differences in the number of connections). **Top:** Following task performance the number of short distances significantly increased and the number of longer distance connections significantly decreased at all cost levels. Significance is indicated by the grey patches at the top of the figure (see File S2 for a 2D version of this plot). Note that graphs at higher cost levels comprise the edges from graphs at lower cost levels which lead to larger differences in the number of connections at higher cost levels (ΔN). **Bottom:** Distance changes at 50% cost level: impaired subjects (red) showed a more pronounced (but not significant) shift towards shorter connections following the task as compared to more resilient subjects (blue).

For physical distances, the analyses of the distribution of the physical lengths of the connected regions (edges) showed a significant shift from long distance connections (distances in the range from 78 to 108 mm) towards shorter distance connections (distances in the range from 8 to 53 mm) following task processing (from RS1 to RS2; Figure 5 D). This shift was present at all investigated cost levels (Figure 5 D, top panel).

*Performance related effects.* The individual vigilance decrement as measure of individual performance correlated significantly with the mean connectivity strength (“main effect of performance”, see Table 1). Furthermore, changes in connectivity strength and clustering between resting state periods (R1 vs. RS2 and R2 vs. RS3) were significant different for subjects with different performance levels (“interaction RS*performance”, see Table 1). On post-hoc level, performance related effects of connectivity strength and clustering were only significant during the post-task phase (RS2 and RS3, Table 2, Figure 4 A and 4 C). The change in connectivity strength and clustering from RS2 to RS3 correlated with individual performance (Figure 5 A and 5 C, Table 2), but not the change from RS1 to RS2.

For both metrics, attentionally resilient subjects (subjects with better performance) showed a significant reduction in connectivity strength and clustering in the last RS period (RS3) in the direction of pre-task values. For attentionally impaired subjects (subjects with significant reaction time increase during task performance) connectivity strength and clustering showed no such recovery effects; their numerical values further increased until the end of the experiment. This correlation between clustering and performance was significant at all investigated cost levels (see Figure S2.2 in File S2). If performance was investigated as categorical factor (resilient vs. impaired subjects) clustering of behavioural impaired subjects was significantly higher in the cost range from 15 to 50% (see Figure S2.1 A in File S2). The aforementioned shift in the physical distance distribution towards more short range connections following task performance was further pronounced (but not significant) for subjects with higher vigilance decrements (Figure 5 D, bottom panel).

A further interesting finding is that the connectivity strength of RS1 (RS period before the task has started) showed a trend for a significant correlation with subsequent task performance (Pearson’s r = 0.43, p = 0.06, see also File S3). The observation that higher levels of connectivity strength were accompanied by higher vigilance decrements were later confirmed for the levels of connectivity strength *during* the task (see below).

**Figure 6:**
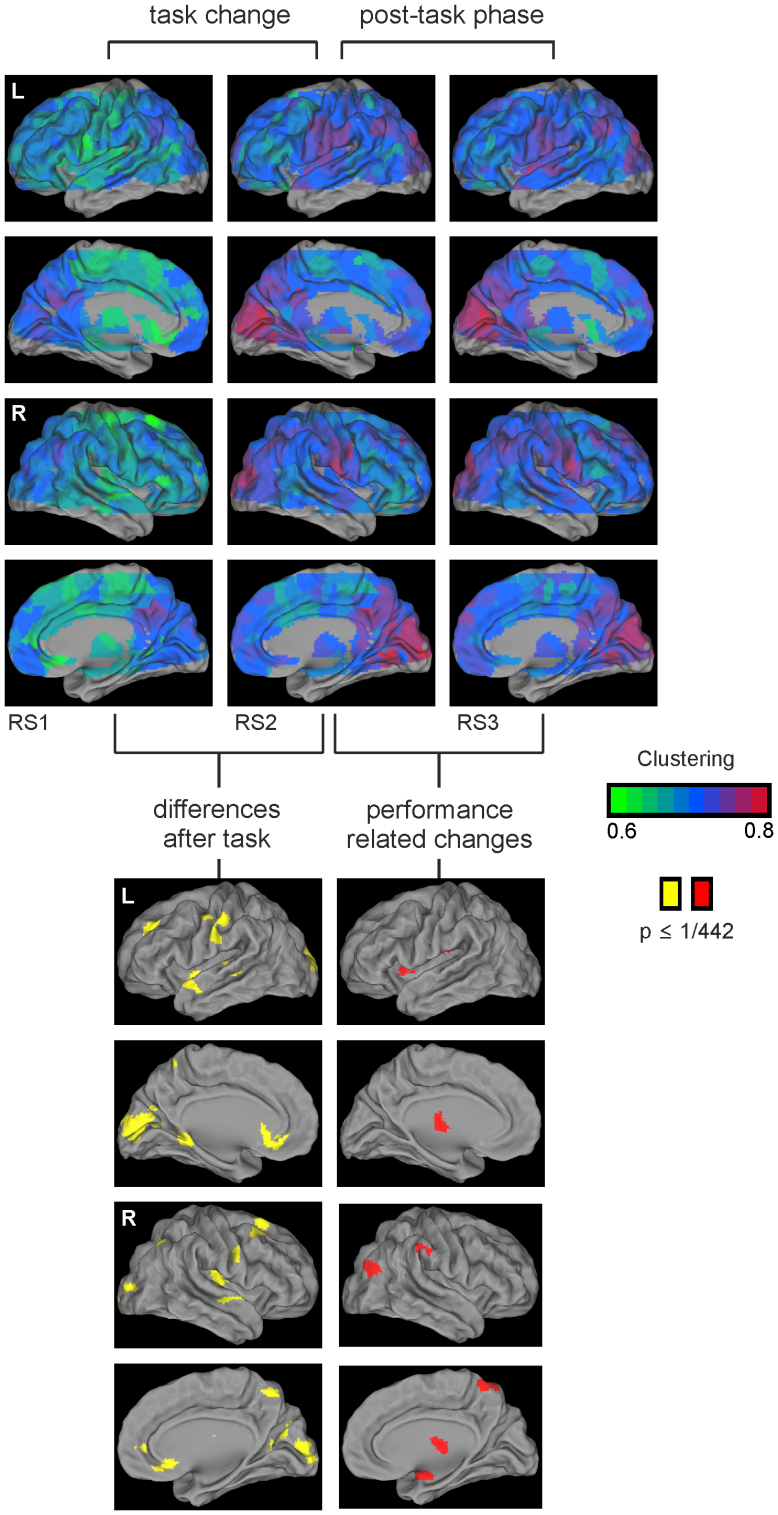
Changes in nodal network clustering over resting state fMRI periods. **Top:** Clustering changed over the three resting state fMRI periods. Following task performance all brain regions show higher clustering or cliquishness. **Bottom left:** Significantly different changes in clustering at nodal level (yellow) were evident among others in visual cortex and basal forebrain; brain areas involved in the processing of visual sustained attention tasks. **Bottom right:** Significant changes in clustering in the post-task phase that were related to vigilance decline were found among others in the thalamus (red).

*Effects on nodal level.* We also investigated the effects of rest period on topological metrics at the level of nodes or brain regions. As shown in Figure 6 we found major increases in clustering following task processing (from RS1 to RS2) in areas including the primary visual cortex the superior temporal gyri, the superior frontal gyri, the cuneus/medial parietal cortex, the left primary sensory cortex, the left lingual gyrus, the basal forebrain, and the anterior cingulate cortex.

Again, individual performance showed regional correlations with changes at nodal level only during the post-task phase (i.e. between RS2 and RS3). Regions that correlated with individual performance were localized in the left and right thalamus, right supramarginal gyrus, right precuneus, right amygdala, left insular cortex and right middle occipital cortex (see Figure S2.3 and S2.4 in File S2 for an analysis of nodal effects as a function of cost level).

#### 2.3. Changes during task periods (T1 to T5)

The data analysis showed significant effects of the continuous factor “task period” on all of the investigated metrics (Table 3). During the task, connectivity strength and clustering continuously increased with task progress, global efficiency and physical distances showed the opposite development and continuously decreased with the task progress (Figure 4, periods T1 to T5).

**Table 3:** Mixed-effects analysis of task data: Effects of time-on-task on (i) mean connectivity strength, (ii) functional network and (iii) physical distance metrics.

Factor	Statistics
Connectivity strength(a)	**T(79) = 2.19, p<0.05**
Clustering	**T (79) = 2.47, p<0.05**
Global Efficiency(a)	**T (79) = −3.55, p<.0001**
Physical distances(a)	**T (79) = −2.32, p<0.05**

(a) corrected for unequal variances.

Similar to the changes in the RS periods, the changes of the graph metrics during the task were more pronounced at lower cost levels (e.g. at 10% cost level, see Figure S2.1 B in File S2), and were reduced (or became non-significant) for higher cost levels (e.g. at 30% cost level for global efficiency and clustering, Figure S2.1 B in File S2, right panel). The effects of physical distances were stable across all cost levels.

An additional observation was that the connectivity strength during the task was numerically different between the two performance groups (impaired vs. resilient subjects, Figure 4 A). Correlation testing confirmed this observation and revealed that individual vigilance decrements significantly correlated with the level of connectivity strength over all task periods (averaged connectivity strength of all five task periods with vigilance decrements: r = 0.45, p<0.05). Subjects with poorer performance showed higher correlation values during the task.

#### 2.4. Effects of ‘task switch’ (R1, T1, T5 and R2)

The analyses of the changes between RS and task periods revealed a significant effect of the factor “time point” (resting state and task data at the first switch from rest to task vs. data at the second switch from task to rest) for all investigated metrics (Table 4). In other words, similar to the changes identified from RS1 to RS2, connectivity strength and clustering were significantly increased at the task switch at the end of the task (task period 5 and RS2) as compared to the first task switch (RS1 and task period 1) (Figure 4 A and 4 D, Table 5). Global efficiency and physical distances showed again opposite effects and had significantly reduced values at the task switch at the end of the task (Figure 4 B and 4 C, Table 5) as compared to the first task switch.

**Table 4:** Mixed-effects analysis of resting state and task data before and following task switches (R1, T1, T5 and R2): Effects of task switch (RS vs. task period), time point (data at the first switch vs. the second) and task switch-by-time point interaction on (i) mean connectivity strength, (ii) functional network and (iii) physical distance metrics.

Response	Factor	Statistics
Connectivity Strength(a)	Task switch (RS to task, or task to RS period)	F(1,57) = 0.05, p = 0.83
	**Time point (begin or following the task)**	**F(1,57) = 7.52, p<0.01**
	Interaction switch*time point	F(1,57) = 0.26, p = 0.27
Global Efficiency(a)	**Task switch**	**F(1,57) = 60.0, p<.0001**
	**Time point**	**F(1,57) = 25.8, p<.0001**
	Interaction switch*time point	F(1,57) = 3.5, p = 0.07
Clustering	**Task switch**	**F(1,57) = 23.15, p<.0001**
	**Time point**	**F(1,57) = 16.07, p<0.001**
	Interaction switch*time point	F(1,57) = 3.53, p = 0.07
Physical Distances(a)	Task switch	F(1,57) = 0.93, p = 0.34
	**Time point**	**F(1,57) = 21.18, p<.0001**
	Interaction switch*time point	F(1,57) = 0.71, p = 0.40

(a) corrected for unequal variances.

**Table 5:** Pairwise comparisons of resting state and task data before and following task switches (R1, T1, T5 and R2): Effects on (i) mean connectivity strength, (ii) network topology, (iii) physical distances.

	RS1 to T1(a)	T5 to RS2(a)	RS1/T1 to T5/RS2^(a, b)^
Connectivity Strength	T = −0.36, p = 0.72	T = −2.04, p = 0.06	**T = −2.1, p<0.05**
Global Efficiency	**T = −5.91, p<0.0001**	**T = 6.32, p<0.0001**	**T = 4.38, p<0.001**
Clustering	**T = 2.27, p<0.05**	**T = −6.47, p<0.0001**	**T = −3.14, p<0.01**
Physical Distances	T = −0.71, p = 0.48	T = 1.37, p = 0.18	**T = 3.80, p<0.01**

(a) Paired t-tests, df = 19.

(b) T-tests based on averaged values for RS1/T1, respectively T5/RS2.

Furthermore, measures of network topology were significantly affected by the change from resting state towards task processing (factor “task switch”, Table 4): Global efficiency was significantly increased during task processing (first task switch from RS1 to task period 1) and was significantly reduced during rest (second task switch from task period 5 to RS2) (Figure 4 B, Table 5). In contrast, clustering was significantly lower during task processing and significantly higher during rest (Figure 4 C, Table 5).

### 3. Possible Impact of Head Movements of Functional Connectivity

To check for possible effects of head movements we calculated the number of frames that would need to be removed from each dataset by “scrubbing” according to the criteria of Power et al. [45]. We found that only 23/60 (38%) of resting datasets would need more than one frame “scrubbed”. These datasets were similar distributed across ‘attentionally resilient’ and ‘attentionally impaired’ subjects and the percentage of affected datasets did not significantly differ between both subgroups (Χ^2^ = 1.13, df = 1, p = 0.29): 9/30 were in the group of ‘attentionally resilient’ subjects, and 14/30 were in the groups of ‘attentionally impaired’ subjects. Second, we observed that “scrubbing” did not change the relationship between functional connectivity and physical distances when the “unscrubbed” correlation matrix is subtracted from the “scrubbed” correlation matrix (ΔR). Thus, “scrubbing” time points (using the thresholds described in the Power et al. paper) provide no additional benefit to our data (see File S4 for further analyses).

## Discussion

Does task performance have long-lasting effects on functional network topology? Our results revealed (i) that prolonged attentional task performance led to less brain network integration following task processing, (ii) that this reduction in network integration already started during task processing and (iii) that network integration remained diminished for long periods of time. Task performance initially increased network integration, but with ongoing time-on-task functional brain topology became less integrated, less efficient, more clustered and showed less long-distance connections. Following task performance these changes in network integration could be still detected after 6 minutes of resting state.

As a second major finding, we could show that late changes in network topology (more than 6 minutes after the task) were correlated with individual differences in cognitive resilience during task performance. Attentionally resilient participants showed a larger reduction of network clustering and disintegration, i.e., a greater degree of recovery towards pre-task network topology during the post-task period.

### Task Performance Induces Long-term Changes in Functional Brain Network Topology

Several studies reported changes in resting state connectivity in low frequency ranges (<0.1 Hz) subsequent to task performance. Changes in connectivity strength were evident after motor [15], language [16] and memory tasks [46]. Other studies reported that motor [17] and perceptual learning [18], [19] can lead to long-lasting changes in functional connectivity and a modulation of region specific plasticity. Our study confirms and extends these findings. Our results show that task manipulation introduces changes in functional connectivity, but also in intrinsic brain *network topology and configuration.*

As a further new aspect we found that changes in network topology persist for at least 6 minutes following task performance. Long-lasting effects after task performance on RS oscillations have been infrequently reported in studies to date. One study by Barnes et al. [20] could show that endogenous oscillations in different brain regions stay altered for more than 15 minutes after task performance. In contrast to Barnes et al. [20], who investigated isolated time series, our study investigated the functional topology of the whole brain network and showed that effects on functional network topology persist over a long time period. The two RS periods after task processing covered a time window of 12 minutes and functional connectivity, physical distances, global efficiency and clustering were still changed in RS3 (6 min to 12 min after the task).

### Long-lasting Changes in Network Topology are the Result of a Continuous Process during Task Performance

During task performance, two different processes seem to influence functional network topology. As first process, task performance increased network integration and induced higher global efficiency and less clustering. Network integration remained higher during task processing in comparison to resting state periods, but with ongoing time-on-task brain networks showed the opposite effects and became more fragmented with more clustering, and showed less global efficiency and shorter distances between brain nodes. This on-going process of network fragmentation over time was also evident when data around the switch between rest and task condition at the beginning and the end of the experiment were analysed; at the end of the task networks were less efficient, more clustered and showed more short distance connections. In summary, within the current study our results showed that declines in network integration started during task performance, progressed with on-going task performance, and that networks remained fragmented for a long time period.

While measures of network topology were significantly changed from rest towards task processing, ‘whole brain’ functional connectivity seemed to be less sensible towards direct effects of task performance, and showed no general offset with the onset of task performance and continuously increased with on-going time of task.

Only few studies investigated changes in functional network topology *during* task performance (e.g. [11], [47]). One study that described rapid adaption processes of brain topology towards different task conditions was performed by Kitzbichler et al. [14]. They used magnetoencephalography (MEG) to analyze the high frequency oscillations and functional brain networks during the performance of a working memory task with different task demands. Similar to our results, in which the resting state condition can be assumed to be less demanding than the task condition, Kitzbichler et al. [14] found that higher demanding tasks were associated with increased network integration (higher global efficiency and increased connection distance, reduced clustering and reduced modularity). Our data confirmed that higher task demands (in our case from rest to task periods) lead to a significant increase in network integration. Note however that our data were in a much lower frequency range.

Prior data provide evidence that these changes in network integration might be related to cognitive fatigue. In a previous study we measured resting state periods that were presented intermixed with task blocks during which participants performed a sustained attention task [21]. Within this study we found that a decline in behavioural performance, an increase in fatigue ratings and the factor time-on-task were significantly correlated with the reduction in network integration during resting periods. Further support derives from EEG studies which showed a correlation between sleepiness and network disintegration [48].

### Behavioural Performance and Functional Brain Networks

Participants showed longer reaction times at the end of the vigilance task and these behavioural effects were accompanied by reduced brain network integration following task performance. Previous studies support an association between brain topology and task performance. Subjects with higher scores in intelligence tests (based on performance in various tasks) showed shorter path lengths and higher global efficiency values in brain network topology [6], [7]. Further, recent studies investigating aging effects found age-related changes in network topology and showed that aged subjects have decreased global efficiency and increased clustering [10], [11]. From previous behavioural studies it is known that aged subjects show poorer performance in attentional tasks [8], [9]. A more direct piece of evidence for the correlation of network topology and attentional task performance is given by a study from Wang et al. [12]. Wang et al. [12] compared the brain network topology of children with attention deficit hyperactivity disorder (ADHD) and healthy children. Children with the attentional deficit syndrome showed lowered global efficiency and significantly increased clustering in their brain topology. In summary, previous group and correlation studies suggest that higher task performance is related to higher network integration. Our data show that changes in network integration and task performance can also be manipulated experimentally by a challenging attentional task.

Interestingly, mean connectivity strength was significantly correlated with declines of behavioural performance. Reaction times and connectivity strength continuously increased with time-on-task and the mean connectivity strength over all task periods significantly correlated with individual vigilance decrements. In addition, subjects with higher connectivity strength during the resting state period before task performance showed a tendency for higher vigilance decrements (p = 0.06). Thus, subjects already showed differences in brain networks before task performance which might have influenced their cognitive resilience during task processing (see also File S3). A relation between connectivity strength and wakefulness or arousal has been previously suggested [49]–[51]. Larson-Prior et al. [51] reported increased connectivity strengths in the dorsal attention network when subjects descended into light sleep. Thus, the higher level of connectivity strength in subjects that showed poorer performance in our study might reflect lower levels of arousal in these subjects.

With respect to network topology, we found that individual behavioural differences did not predict task induced changes in network topology following the end of the task (i.e. RS1 vs. RS2), but were highly correlated with changes in topology in the post-task or recovery phase (RS2 and RS3). Only few studies have investigated recovery effects after tasks on endogenous dynamics so far. Barnes et al. [20] compared changes in endogenous oscillations on a longer time scale after a task with different workloads. They found workload dependent recovery effects in endogenous oscillations; a low load condition led to a faster and earlier recovery (about 400 s post-task) of the fractal properties of the BOLD time series. In our study we did not vary the workload, but the variability of performance between subjects indicated that the same task demand could introduce large differences in individual cognitive resilience. Brain networks in subjects with high vigilance decrements further continued to show increased global connectivity strength and mean clustering in the RS3 period. In contrast, brain networks in subjects with low or no vigilance decrement revealed a pattern of connectivity and a level of clustering which resembled that prior to task performance. Although the acquired RS time window of 12 minutes post-task was not long enough for a full restoration of all network metrics our study is the first which shows demand-related recovery effects on functional network topology.

### Changes of Clustering on Regional Level

Beside the analysis of the averaged clustering values over all nodes (seed regions of the template), we further investigated regional changes in clustering before and following the task and in the post-task phase. On nodal level, effects of increased clustering immediately following the task (from RS1 to RS2) were, among others, evident bilaterally in the visual cortices, the superior temporal cortices and the basal forebrain (Figure 6, bottom left). Some of these regions including the visual and temporal cortices also showed increases in clustering following a visual attention task in a different set of volunteers [21]. These regions have been previously discussed in the context of visual processing and visual spatial attention (e.g. [52]) and the observed changes in clustering in these regions may reflect general reconfiguration processes following visual attention tasks. More specific related to attentional effort, we found increased clustering after task processing within the region of the basal forebrain. During task performance the activation within this brain region increased with higher cognitive demands due to prolonged time intervals between target stimuli [25]. The cholinergic basal forebrain is a crucial part of the attentional effort network as proposed by Sarter [53] and is suggested to trigger activity in the anterior attention system in medial prefrontal cortex and to optimize processing in sensory cortical regions. Animal evidence indicates that attentional effort increases ACh release in medial prefrontal cortex [54]. In summary, our data provide first hints that task specific recruitment of brain regions can produce region-specific hang-over effects in brain topology after task performance.

Significant correlations between behaviour and nodal clustering were only observed in the post-task phase (RS2 to RS3) and among others evident in the thalamus, a key structure of the arousal system [55], [56]. Attentionally impaired subjects showed further increases of clustering in this region, whereas resilient subjects showed decreases of clustering. These differential effects might be related to the long- and short-lasting cognitive processes elicited by the sustained attention task [25]. During task processing participants had to detect an infrequent colour change of a simple visual stimulus during a time period of more than 30 minutes. Even though there are several factors that affect performance declines during periods of tonic alertness [57], previous studies support the idea that the attention system interacts with arousal level [56]. All subjects might have “used” the same brain networks for attentional processing, but subjects who performed the task in a less efficient way probably showed larger reductions of arousal following task performance. These differences in arousal between subjects may increase after a longer time period of resting state (resting state period 2 and 3) in which participants have to refrain from falling asleep. It is a common finding in psychology that inter-individual differences are more evident in “weak” task conditions in which less cues provide behavioural psychological pressure and that are more ambiguously structured to engage a certain behaviour [58].

### Biological Explanations for Long-lasting Effects of Task Performance

Previous studies have shown that learning-induced plasticity can have a long-lasting impact on subsequent RS periods [17]–[19] and might therefore be responsible for the task-induced changes in functional connectivity and network topology observed after our sustained attention task. Animal data suggest that prolonged effort in attention tasks increases the release of acetylcholine (ACh) in prefrontal brain regions resulting in a top-down adjustment of sensory processes [53]. ACh induced plasticity can last several minutes even though ACh concentration has reached baseline level again (for a review see [59]). Hence, changes in resting state connectivity after performance of a sustained attention task may be the consequence of prior task-induced ACh release.

On molecular level metabolic costs may account for long-lasting effects of task performance. Mental tasks are accompanied by an increased level of glycolysis in the brain [60], [61]. Raised levels of glycolysis can last up to 40 minutes after termination of task performance [62] and prolonged aerobic glycolysis can cause imbalance in cell metabolism (e.g. proton and lactate accumulation [63]). Our data revealed that the distribution of the physical distance between functionally connected nodes was shifted in favour of shorter distances after task performance. Neuronal transmission over shorter physical distances was earlier described as one strategy of the brain in order to preserve metabolic costs [64].

### Methodological Considerations

Most graph analyses ignore that correlations between two brain regions can also be influenced by a common covariate that induces indirect correlations. Even though indirect correlations are a general problem that also affects the analyses of resting state data they become more obvious during the analysis of task data in which the experimental stimulation might have simultaneously influenced two brain regions. Therefore we focussed in the interpretation of our results mainly on changes between conditions with the same experimental stimulation (comparisons between rest conditions or within task blocks).

Even though previous results revealed that functional connectivity do not significantly change if participants are instructed to fixate or to keep their eyes open without fixation over a time period of 5 minutes [65], future studies might investigate whether physical fatigue of the eyes influences network integration.

### Implications of Long-lasting Changes in Network Topology following Tasks

In many studies investigating neural correlates of task-related processing (e.g., in fMRI, PET or ERP studies) spontaneous signal fluctuations were only considered as noise factor. Over the last years this perspective has changed, since previous studies have shown that, for example, spontaneous blood oxygen level dependent (BOLD) signal fluctuations contribute to the prediction of variability in behavioural performance within and between subjects [22], [66]–[68]. In computational neuroscience, previous results revealed that the complex network architecture of brains rapidly changes to meet the requirements of specific task demands [14], [69]. As first study, our results show that task performance has also reflexive effects and leads to long-lasting changes in endogenous brain networks after task performance. Our data show that these long-lasting effects on brain network topology are correlated with behavioural measures of cognitive resilience in a prior sustained attentional task. Future studies, with several task and RS periods within one fMRI scanning session, might also show that individual differences in network recovery can be used to predict differences in behavioural performance within following task periods.

In real world, economical reasons often force researchers to present several tasks within one fMRI scanning or MEG session, neglecting or underestimating changes in functional brain networks resulting from a previous task. Our data indicate that 12 minutes of rest period between each task are not enough to fully recover functional brain networks from previous task processing. Our data strongly encourage to take into account “hang-over” effects on the organization of post-task brain networks that possibly interact with the processing of new tasks. These “hang-over” effects introduce a specific brain state, which will impact on subsequent neural processing and behavioural performance. In future, the individual capability of brain network recovery might be an additional measure to obtain cognitive resilience of patients and to detect early symptoms of potential neurological disorders. Our data support that the recovery of connectivity strength and network topology may serve as biomarkers of cognitive demand and resilience that significantly contribute to the understanding of inter-subject differences in the recovery from challenging tasks.

## Supporting Information

File S1 Alternative parcellation templates.
(PDF)

File S2 Graph metrics at different cost levels.
(PDF)

File S3 Pre-task level on connectivity strength and performance.
(PDF)

File S4 Analyses of suspect motion-corrupted frames.
(PDF)
