# Supplementary material for: Long-Term Effects of Attentional Performance on Functional Brain Network Topology
Source: PLoS One. 2013 Sep 9;8(9):e74125. doi: 10.1371/journal.pone.0074125 (PMC3767656; doi:10.1371/journal.pone.0074125)
Supplement: File S1 — Alternative parcellation templates. (PDF) [file pone.0074125.s001.pdf]

SUPPORTING INFORMATION FILE S1 – ALTERNATIVE PARCELLATION TEMPLATES

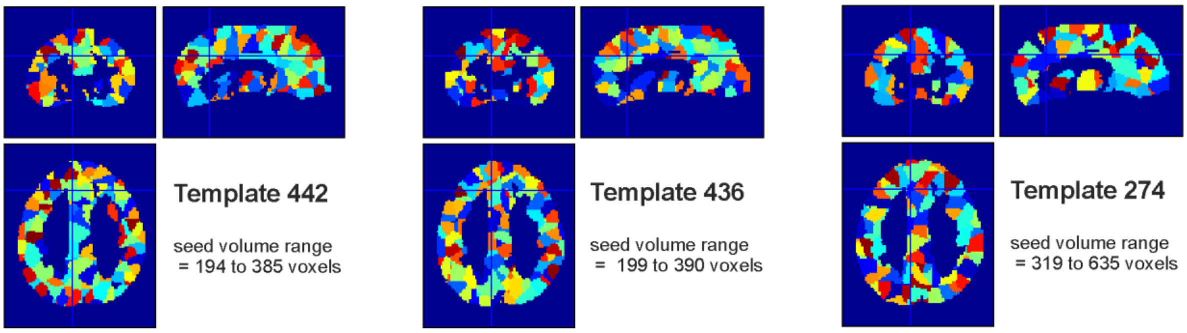


SUPPORTING FIGURE S1.1. **Different randomly parcellated templates lead to similar results for the RS analysis** (see table below). **Left:** Parcellation template with 442 seed regions as used in the main manuscript. **Middle:** Alternative template with the similar number (436) and similar sizes of seed regions as in the main manuscript. **Right:** Coarser sampled template with 274 seed regions.

SUPPORTING TABLE S1.1. Main statistics (LME) of RS periods (changes following the task and in the post-task phase) for all three templates.

| | | Template 442 (same as main results table 1) | | Template 436 | | Template 274 | |
|---|---|---|---|---|---|---|---|
| Response | Factor | | | F value | | F value | |
| Connectivity Strength | **Resting State (RS)** | **F(2,36) = 5.80,** | **p < 0.01** | **5.74** | **p < 0.01** | **5.17** | **p < 0.05** |
| | **Performance** | **F(1,18) = 8.46,** | **p < 0.01** | **8.37** | **p < 0.01** | **8.33** | **p < 0.01** |
| | **Interaction RS*Perf.** | **F(2,36) = 4.32,** | **p < 0.05** | **4.23** | **p < 0.05** | **3.79** | **p < 0.05** |
| Global Efficiency | **RS** | **F(2,36) = 15.8,** | **p < .0001** | **19.2** | **p < .0001** | **14.7** | **p < .0001** |
| | Performance | F(1,18) = 0.1, | p = 0.74 | 0 | p = 0.93 | 0 | p = 0.92 |
| | Interaction RS*Perf. | F(2,36) = 1.6, | p = 0.22 | 1.6 | p = 0.21 | 1.2 | p = 0.31 |
| Clustering | **RS** | **F(2,36) = 8.35,** | **p < 0.01** | **8.95** | **p < 0.001** | **9.1** | **p < 0.001** |
| | Performance | F(1,18) = 2.59, | p = 0.13 | 1.98 | p = 0.18 | 2.22 | p = 0.15 |
| | **Interaction RS*Perf.** | **F(2,36) = 6.18,** | **p < 0.01** | **4.34** | **p < 0.05** | **1.89** | **p = 0.17** |
| Physical Distances | **RS** | **F(2,36) = 22.42,** | **p < 0.001** | **16.81** | **p < .0001** | **16.43** | **p < .0001** |
| | Performance | F(1,18) = 0.24, | p = 0.63 | 0 | p = 0.97 | 0 | p = 0.97 |
| | Interaction RS*Perf. | F(2,36) = 1.82, | p = 0.17 | 2.39 | p = 0.11 | 1.96 | p = 0.16 |

The main effects described in the manuscript were widely preserved when using alternate templates, even though if the number of seed regions were significantly reduced as in template 274. For clustering the interaction of resting state and performance is not significant for template 274 (indicated in red). However, in the post-hoc test the differences in clustering between RS3 and RS2 were significantly correlated with performance (Clustering of RS3-RS2: correlation with individual vigilance decrement with Pearson’s r = 0.6, p<0.01 and dichotomous testing for both performance groups (impaired vs. resilient subjects), two-sample t-test: t(18) = 3.09, p<0.01).
