# Supplementary material for: Long-Term Effects of Attentional Performance on Functional Brain Network Topology
Source: PLoS One. 2013 Sep 9;8(9):e74125. doi: 10.1371/journal.pone.0074125 (PMC3767656; doi:10.1371/journal.pone.0074125)
Supplement: File S2 — Graph metrics at different cost levels. (PDF) [file pone.0074125.s002.pdf]

SUPPORTING INFORMATION FILE S2 – GRAPH METRICS AT DIFFERENT COST LEVELS

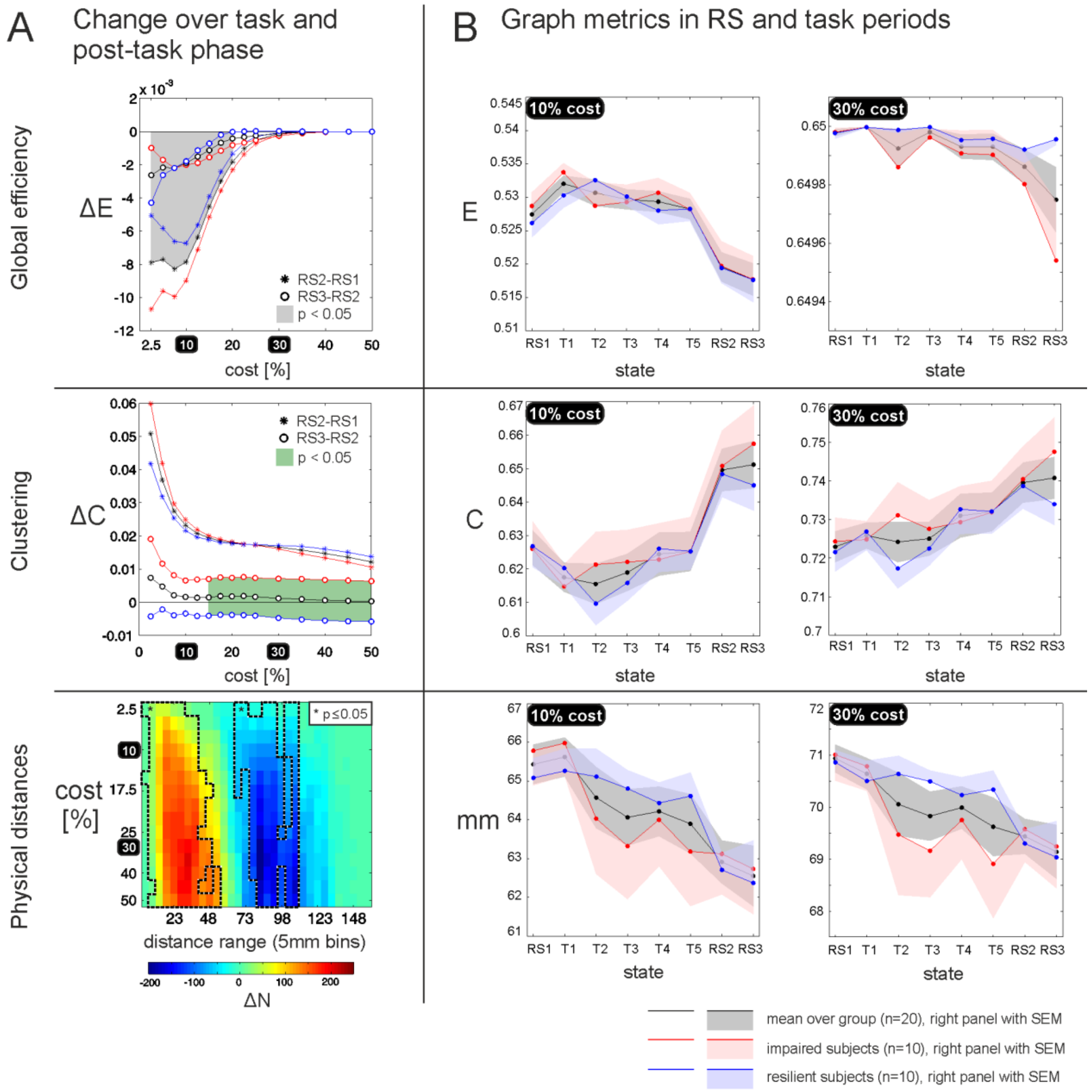

SUPPORTING FIGURE S2.1: **Global efficiency (E), clustering (C) and physical distances at different cost levels. A, top panel:** *Changes in global efficiency following task performance.* Changes in global efficiency from RS1 to RS2 were more pronounced in the lower cost range. If averaged over attentionally resilient and impaired subjects (black curve) global efficiency significantly decreased following task performance in the range from 2.5% to 20% (grey area, p<0.05). At all cost levels differences between both performance groups were not significant (between the blue and red curves). *Changes in global efficiency in the post-task period:* Changes of global efficiency from RS2 to RS3 were not significant at any cost level. Impaired and resilient subjects showed similar effects. **Middle panel:** *Changes in clustering following task performance:* A significant change in clustering following task processing (RS1 to RS2) was found at all cost levels (more pronounced at lower cost levels). Attentionally resilient and impaired subjects showed similar results (blue vs. red). *Changes in clustering in the post-task period*: In the post-task phase clustering of the entire group (black) did not change from RS2 to RS3. However, significant differences in clustering were found between resilient and impaired subjects in the cost range from 15% to 50% (green area, p<0.05). **Bottom Panel:** Change of physical distance distribution of edges for all costs (two-dimensional version of Figure 5 D of the main text). N = number of edges; at each cost level tests were Bonferroni corrected with p*=p/32 for the 32 tests at each distance bin, mm=millimeter.

B, left panel. *At the single cost level of 10%* the overall pattern as well as the significance of the effects as described in main manuscript were preserved. **Right panel:** *At the single cost level of 30%* the main effects with regard to the RS periods (changes from R1 to R2 and post-task development) were persevered; effects of ‘task switch’ for global efficiency and clustering were not significant anymore.

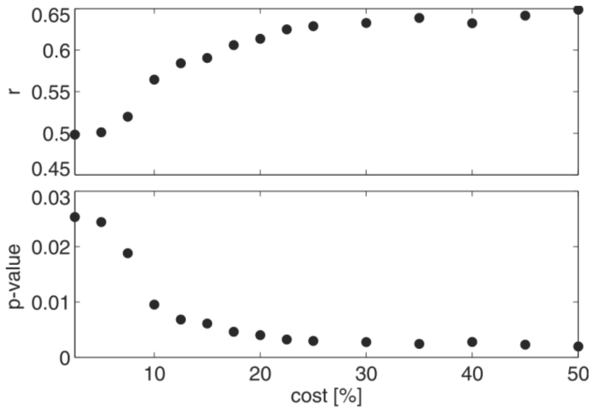

SUPPORTING FIGURE S2.2: **Correlation of individual vigilance decrements with post-task change (RS3-RS2) in clustering over cost levels.** The correlations of individual vigilance decrements with post-task clustering changes (RS2 to RS3) were significant at *all* cost-levels. Significance level first decreased due to the higher number of edges within the graph and became stable at 20 percent of costs.

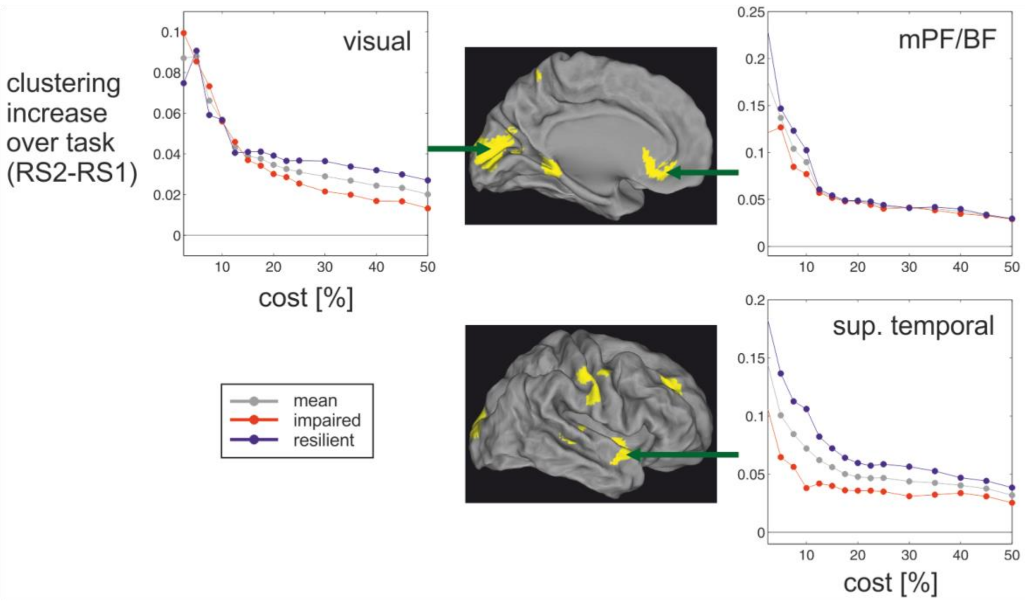

SUPPORTIONG FIGURE S2.3: **Changes in nodal clustering following task processing (RS1 vs. RS2) separated for cost levels.** Selected regions. Effects of task processing on clustering were analyzed as a function of cost level. Within those brain regions discussed within the main text (visual areas, medial prefrontal region/basal forebrain (mPF/BF), and superior temporal gyrus) effects on nodal clustering were mainly driven by differences between graphs within the low-cost-range with lower connection densities. Graphs within the low cost range have on average higher correlations values and are less influenced by noisy connections.

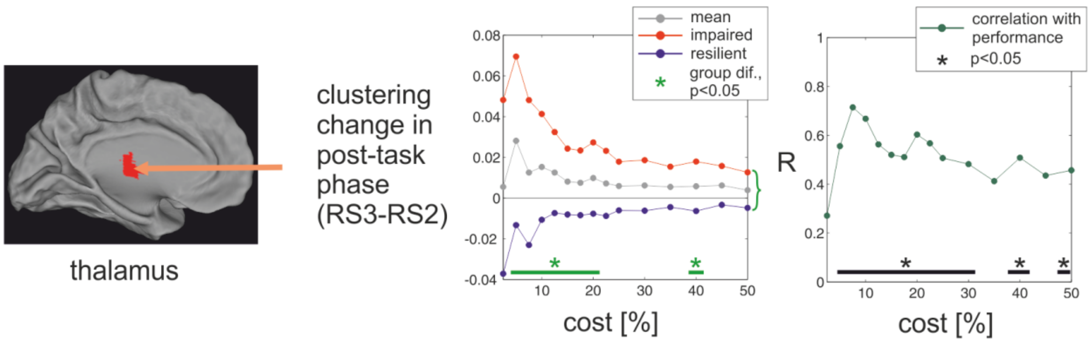

SUPPORTING FIGURE S2.4: **Changes in nodal clustering in the post-task phase (RS2 vs. RS3) plotted over costs and its correlation with performance.** Over all subjects, the thalamus (left panel) did not show any significant changes in clustering during the post-task phase. However, changes in clustering were opposite for resilient and impaired subjects with stronger differences in the low cost range (middle panel). This result was also supported by correlation analysis: the individual vigilance decrements and the change in clustering in the post-task period correlated over a wide cost range and correlation values tended to be higher for the low cost range (right panel). Graphs within the low cost range have on average higher correlations values and are less influenced by noisy connections.
