# Supplementary material for: Long-Term Effects of Attentional Performance on Functional Brain Network Topology
Source: PLoS One. 2013 Sep 9;8(9):e74125. doi: 10.1371/journal.pone.0074125 (PMC3767656; doi:10.1371/journal.pone.0074125)
Supplement: File S3 — Pre-task level on connectivity strength and performance. (PDF) [file pone.0074125.s003.pdf]

SUPPORTING INFORMATION FILE S3 – PRE-TASK LEVEL OF CONNECTIVITY STRENGTH AND PERFORMANCE

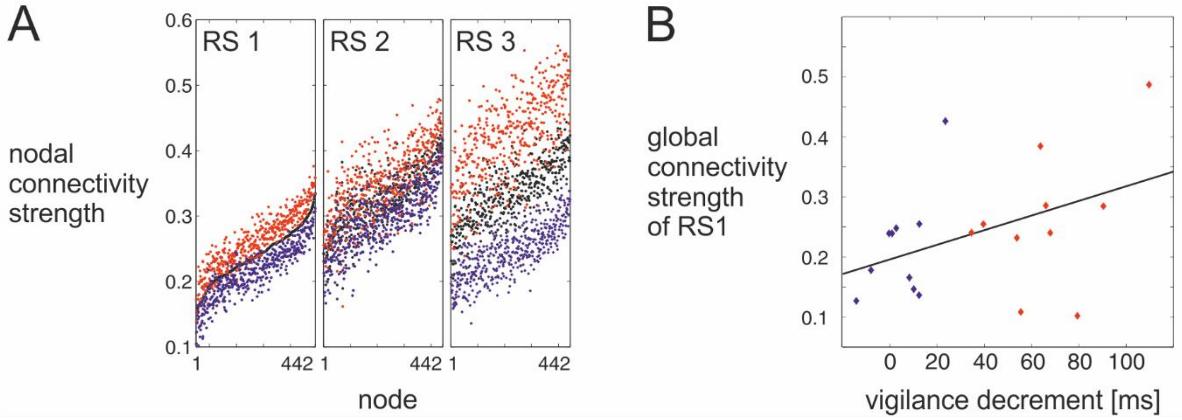

SUPPORTING FIGURE S3.1. **(A)** Nodal connectivity (i) averaged over all subjects (black dots), (ii) averaged over the subgroup of "attentionally impaired" subjects (red dots), and (iii) averaged over the subgroup of "attentionally resilient" subjects (blue dots) are shown. The nodal connectivity of a certain node is the mean functional connectivity between this node and all other nodes within the network. Attentionally impaired subjects (red) as compared to attentional resilient subjects (blue) showed higher nodal connectivity strengths already before the task has started (RS1). (**B**) Subjects with increased global connectivity before task performance (averaged across nodes) showed larger vigilance decrements in the following sustained attention task ($r = 0.43$, $p = 0.06$). This tendency was preserved when using alternative templates (see Supplemental Information in File S1).
