# Supplementary material for: Long-Term Effects of Attentional Performance on Functional Brain Network Topology
Source: PLoS One. 2013 Sep 9;8(9):e74125. doi: 10.1371/journal.pone.0074125 (PMC3767656; doi:10.1371/journal.pone.0074125)
Supplement: File S4 — Analyses of suspect motion-corrupted frames. (PDF) [file pone.0074125.s004.pdf]

SUPPORTING INFORMATION FILE S4 – ANALYSES OF SUSPECT MOTION-CORRUPTED FRAMES

We have analyzed all datasets presented in this paper fully for motion-related effects. In accordance with Power *et al. (2012)* we have computed a number of metrics to identify suspect motion-corrupted frames of data and scrubbed these frames from our datasets to determine whether this confers any additional benefit. We have computed the following:

1) Framewise Displacement (FD, in mm) – this is the sum of the absolute derivatives of the six motion parameters (as described in Power *et al*., 2012). Rotational parameters were converted to displacements by computing the arc length displacement at the surface of a sphere of radius 50 mm.

2) The DVARS (in %x10) on the time series used for graph analysis. This was computed as the root mean square on the voxel-wise derivatives of percent signal change. This was expressed as %x10 as in Power *et al.* (2012).

3) The number of suspect frames that would be censored by the Power e*t al*. (2012) “scrubbing” methodology. Suspect frames were identified by having either FD > 0.5 mm or DVARS > 0.5 %. These thresholds were identical to those used in Power *et al.* (2012).

4) A parallel set of times series for all datasets where the suspect frames in 3) were “scrubbed” and temporally concatenated in accordance with Power *et al.* (2012). Frames were “scrubbed” after confound regression and temporal wavelet filtering (Carp, 2011).

5) The relationship between ΔR caused by motion scrubbing (simply the “scrubbed” correlation matrix minus the “unscrubbed” correlation matrix) and the Euclidean distance between pairwise correlations of regional time series. We first computed this at a single-subject level, and subsequently averaged ΔR values across subjects for the group-level analysis, as described by Power *et al.* (2012).

6) The r^2 coefficient of determination for each group-level scatter plot between ΔR and Euclidean distance. This was computed using the following formula:

$$R^2 = 1 - \frac{\Sigma(y_i - f_i)^2}{(n-1)\cdot(y_i - \bar{y})^2}$$

where f_i is the linear fit of the scatter plot and n is the total number of pairwise ΔR values.

First, we calculated the number of suspect frames of data that would be removed from the regional time series by “scrubbing”. We found that only 23/60 (38%) of datasets would need more than one frame “scrubbed”. Of these datasets, 9/30 are in the ‘Attentionally Resilient’ condition, and 14/30 are in the ‘Attentionally Impaired’ condition. The total number of frames that would need to be removed from each dataset are shown in Table 1.

Next, we compared the percentage of suspect frames (as a percentage of the total number of frames in each dataset), as a measure of the amount of motion in our datasets, across the two conditions, and across the different resting state scans (Figure 2.1). There is no notable difference in motion between the two conditions ('Attentionally Resilient' and 'Attentionally Impaired'), and whilst there is a slight increase in head movement through the resting state scans (as one would expect), this is not restricted to one group. In addition, this slight increase in movement across resting state scans appears to have no distance-dependent impact on correlation between regional time series (Figure 2.2). To test this last point, we assessed whether there are any distance-dependent biases in correlation strength between regional time series in our data. For this, we scrubbed all datasets and computed the ΔR (which is the "scrubbed" minus "unscrubbed" correlation matrices). These values were plotted against the Euclidean distance between pairwise combinations of time series. We observed no differential effects of correlation strength at long versus short distances, which suggests that "scrubbing" provides no additional benefit to our data (Figure 2.2). To complement this assertion, we computed the r^2 coefficient of determination for each group analysed in this way. The fraction of variance explained by linear fits for our data is negligible (see Figure 2.2) compared to values presented in Power *et al.*, 2012 ($r^2 = 0.18$), confirming our conclusion that there is no relationship between movement-related changes in pairwise correlations between regional time series and distance in our data.

TABLE S4.1: Number of suspect frames per dataset that would need to be "scrubbed" from the time series using the criteria outlined in Power *et al.* (2012) (FD > 0.5 mm or DVARS > 0.5 %). The total number of frames of data in each dataset is 256.

Attentionally Impaired				Attentionally Resilient			
SUBJECT	Rest 1	Rest 2	Rest 3	SUBJECT	Rest 1	Rest 2	Rest 3
001	1	5	9	003	0	0	0
002	0	0	0	004	1	1	0
005	1	0	1	006	1	4	1
008	1	4	1	007	50	24	44
009	0	2	7	011	0	0	0
010	3	21	29	015	2	3	0
012	1	15	12	016	0	0	0
013	7	56	55	017	0	0	0
014	0	0	0	018	2	7	15
020	0	0	5	019	0	0	0

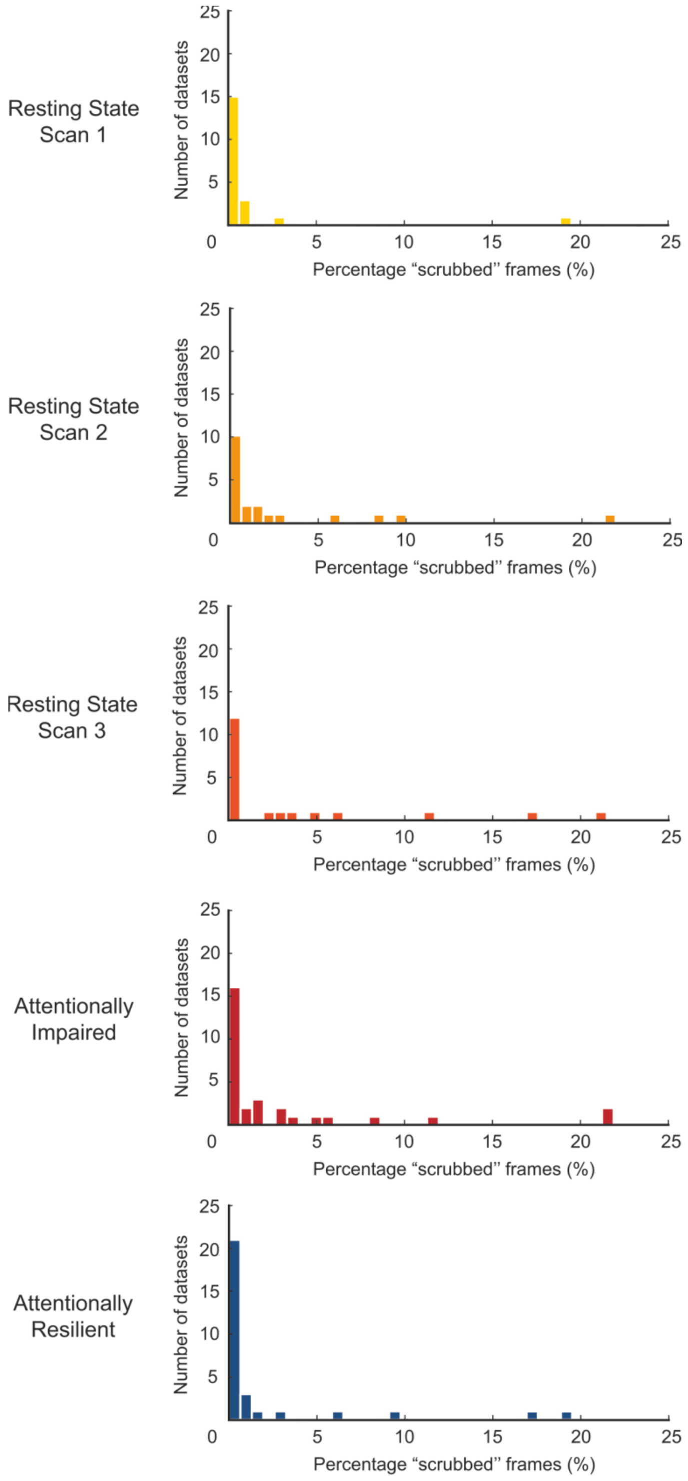

SUPPORTING FIGURE S4.1: Histograms representing the percentage of motion-affected frames in each dataset. Frames are identified using thresholds defined in Power *et al.*, 2012 (frames where FD > 0.5 mm or DVARS > 0.5 %; see METHODS). The histograms labelled ‘Resting state scan’ combine datasets from both conditions (‘Attentionally Resilient’ and Attentionally Impaired’), and similarly, histograms for the two conditions combine datasets from all resting state scans undertaken by subjects in those conditions.

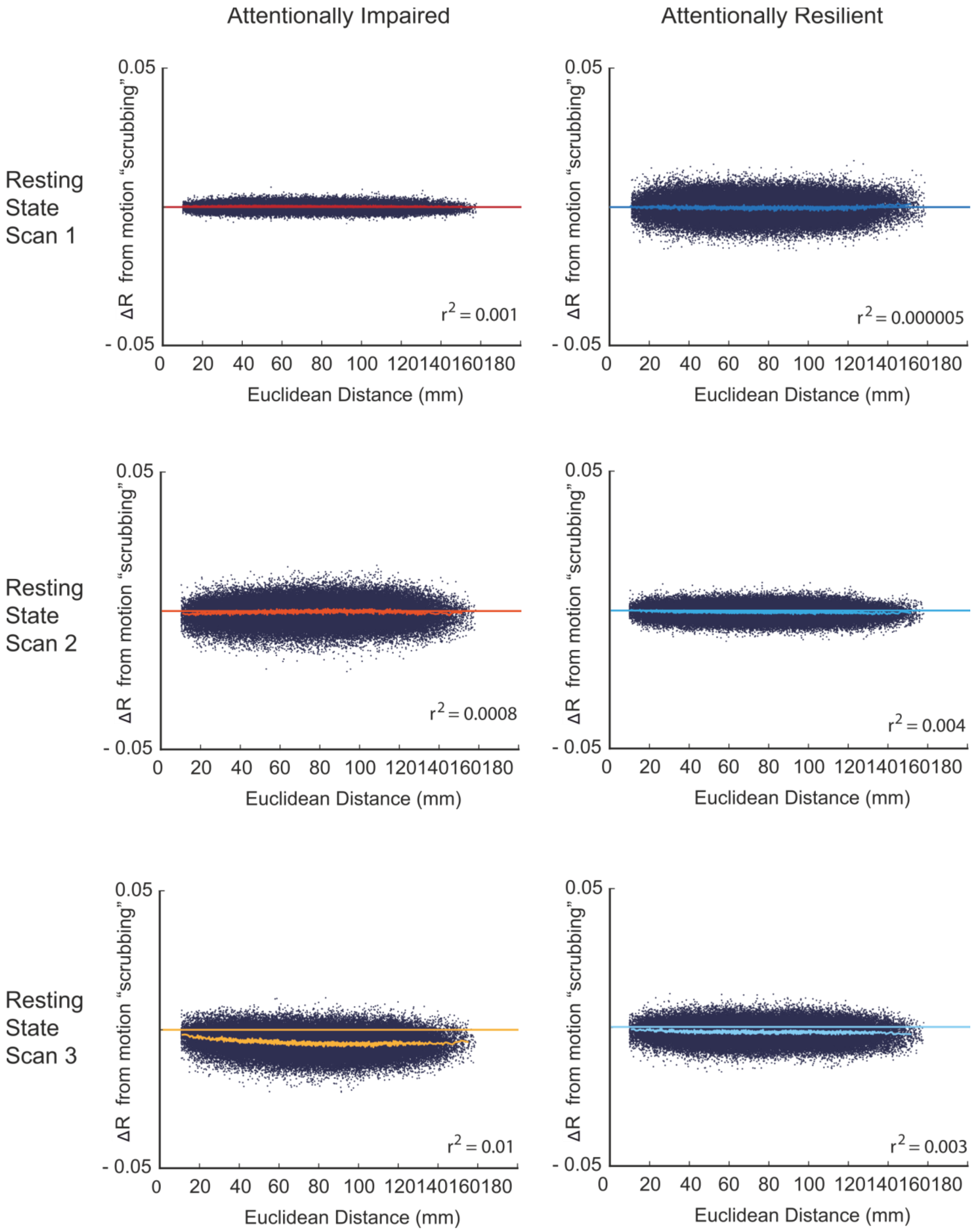

SUPPORTING FIGURE S4.2: Scatter plots showing the impact of time frame "scrubbing" on the relationship between Euclidean distance between regions and ΔR. ΔR is the difference in pairwise correlations before and after scrubbing, and is calculated by subtracting the "unscrubbed" correlation matrix from the "scrubbed" correlation matrix. Each scatter plot was fitted to a moving average line by binning every 100 points (sorted as a function of Euclidean distance) and calculating the average ΔR value for this set of distances. The r^2 values (coefficient of determination) for the relationship between Euclidean distance and ΔR were calculated for each scatter plot and noted alongside the plot (also see METHODS).

References:

Carp, J. (2011) Optimizing the order of operations for movement scrubbing: Comment on Power et al. *NeuroImage*.

Power, J.D., Barnes, K.A., Snyder, A.Z., Schlaggar, B.L. & Petersen, S.E. (2012) Spurious but systematic correlations in functional connectivity MRI networks arise from subject motion. *NeuroImage*, **59**, 2142-2154.
